# A polymorphism in the base excision repair gene *PARP2* is associated with differential prognosis by chemotherapy among postmenopausal breast cancer patients

**DOI:** 10.1186/s12885-015-1957-7

**Published:** 2015-12-16

**Authors:** Petra Seibold, Peter Schmezer, Sabine Behrens, Kyriaki Michailidou, Manjeet K. Bolla, Qin Wang, Dieter Flesch-Janys, Heli Nevanlinna, Rainer Fagerholm, Kristiina Aittomäki, Carl Blomqvist, Sara Margolin, Arto Mannermaa, Vesa Kataja, Veli-Matti Kosma, Jaana M. Hartikainen, Diether Lambrechts, Hans Wildiers, Vessela Kristensen, Grethe Grenaker Alnæs, Silje Nord, Anne-Lise Borresen-Dale, Maartje J. Hooning, Antoinette Hollestelle, Agnes Jager, Caroline Seynaeve, Jingmei Li, Jianjun Liu, Keith Humphreys, Alison M. Dunning, Valerie Rhenius, Mitul Shah, Maria Kabisch, Diana Torres, Hans-Ulrich Ulmer, Ute Hamann, Joellen M. Schildkraut, Kristen S. Purrington, Fergus J. Couch, Per Hall, Paul Pharoah, Doug F. Easton, Marjanka K. Schmidt, Jenny Chang-Claude, Odilia Popanda

**Affiliations:** 1 Division of Cancer Epidemiology, German Cancer Research Center (DKFZ), Heidelberg, Germany; 2 Division of Epigenomics and Cancer Risk Factors, German Cancer Research Center (DKFZ), Im Neuenheimer Feld 280, 69124 Heidelberg, Germany; 3 Department of Public Health and Primary Care, Centre for Cancer Genetic Epidemiology, University of Cambridge, Cambridge, UK; 4 Department of Cancer Epidemiology/Clinical Cancer Registry, University Cancer Center Hamburg (UCCH), Hamburg, Germany; 5 Department of Medical Biometrics and Epidemiology, University Medical Center Hamburg-Eppendorf, Hamburg, Germany; 6 Department of Obstetrics and Gynecology, University of Helsinki and Helsinki University Central Hospital, Helsinki, Finland; 7 Department of Clinical Genetics, University of Helsinki and Helsinki University Central Hospital, Helsinki, Finland; 8 Department of Oncology, University of Helsinki and Helsinki University Central Hospital, Helsinki, Finland; 9 Department of Oncology - Pathology, Karolinska Institutet, Stockholm, Sweden; 10 School of Medicine, Institute of Clinical Medicine, Pathology and Forensic Medicine, University of Eastern Finland, Kuopio, Finland; 11 Cancer Center of Eastern Finland, University of Eastern Finland, Kuopio, Finland; 12 Imaging Center, Department of Clinical Pathology, Kuopio University Hospital, Kuopio, Finland; 13 Central Finland Health Care District, Jyväskylä Central Hospital, Jyväskylä, Finland; 14 Vesalius Research Center (VRC), VIB, Leuven, Belgium; 15 Department of Oncology, Laboratory for Translational Genetics, University of Leuven, Leuven, Belgium; 16 Department of General Medical Oncology, Multidisciplinary Breast Center, University Hospitals Leuven, Leuven, Belgium; 17 Department of Cancer Genetics, Institute for Cancer Research, Oslo University Hospital, Radiumhospitalet, Oslo, Norway; 18 Institute of Clinical Medicine, K.G. Jebsen Center for Breast Cancer Research, Faculty of Medicine, University of Oslo (UiO), Oslo, Norway; 19 Department of Clinical Molecular Biology (EpiGen), Akershus University Hospital, University of Oslo (UiO), Oslo, Norway; 20 Department of Medical Oncology, Erasmus MC Cancer Institute, Rotterdam, The Netherlands; 21 Human Genetics Division, Genome Institute of Singapore, Singapore, Singapore; 22 Department of Medical Epidemiology and Biostatistics, Karolinska Institutet, Stockholm, Sweden; 23 Department of Oncology, Public Health and Primary Care, Centre for Cancer Genetic Epidemiology, University of Cambridge, Cambridge, UK; 24 Molecular Genetics of Breast Cancer, German Cancer Research Center (DKFZ), Heidelberg, Germany; 25 Institute of Human Genetics, Pontificia Universidad Javeriana, Bogota, Colombia; 26 Frauenklinik der Stadtklinik Baden-Baden, Baden-Baden, Germany; 27 Department of Community and Family Medicine, Duke University Medical Center, Durham, North Carolina USA; 28 Department of Oncology, Wayne State University School of Medicine and Karmanos Cancer Institute, Detroit, Michigan USA; 29 Department of Laboratory Medicine and Pathology, Mayo Clinic, Rochester, Michigan USA; 30 Netherlands Cancer Institute, Antoni van Leeuwenhoek Hospital, Amsterdam, The Netherlands

**Keywords:** Survival, Genetic variation, Chemotherapy, Radiotherapy, Anthracyclines

## Abstract

**Background:**

Personalized therapy considering clinical and genetic patient characteristics will further improve breast cancer survival. Two widely used treatments, chemotherapy and radiotherapy, can induce oxidative DNA damage and, if not repaired, cell death. Since base excision repair (BER) activity is specific for oxidative DNA damage, we hypothesized that germline genetic variation in this pathway will affect breast cancer-specific survival depending on treatment.

**Methods:**

We assessed in 1,408 postmenopausal breast cancer patients from the German MARIE study whether cancer specific survival after adjuvant chemotherapy, anthracycline chemotherapy, and radiotherapy is modulated by 127 Single Nucleotide Polymorphisms (SNPs) in 21 BER genes. For SNPs with interaction terms showing *p* < 0.1 (likelihood ratio test) using multivariable Cox proportional hazard analyses, replication in 6,392 patients from nine studies of the Breast Cancer Association Consortium (BCAC) was performed.

**Results:**

rs878156 in *PARP2* showed a differential effect by chemotherapy (*p* = 0.093) and was replicated in BCAC studies (*p* = 0.009; combined analysis *p* = 0.002). Compared to non-carriers, carriers of the variant G allele (minor allele frequency = 0.07) showed better survival after chemotherapy (combined allelic hazard ratio (HR) = 0.75, 95 % 0.53–1.07) and poorer survival when not treated with chemotherapy (HR = 1.42, 95 % 1.08–1.85). A similar effect modification by rs878156 was observed for anthracycline-based chemotherapy in both MARIE and BCAC, with improved survival in carriers (combined allelic HR = 0.73, 95 % CI 0.40–1.32). None of the SNPs showed significant differential effects by radiotherapy.

**Conclusions:**

Our data suggest for the first time that a SNP in *PARP2,* rs878156, may together with other genetic variants modulate cancer specific survival in breast cancer patients depending on chemotherapy. These germline SNPs could contribute towards the design of predictive tests for breast cancer patients.

**Electronic supplementary material:**

The online version of this article (doi:10.1186/s12885-015-1957-7) contains supplementary material, which is available to authorized users.

## Background

Breast cancer ranks among the most important causes of cancer death in women worldwide, but data from recent years reveal that mortality rates are steadily decreasing in Northern European and American countries [1, 2]. This increase in survival can be attributed to both progress in early detection and improved treatment protocols using classical cytostatics and new targeted drugs for estrogen receptor positive tumours and HER2 positive tumours [3, 4]. Current efforts are thus aimed to further advance therapy by developing new drugs but also by considering genetic determinants present in germ line and tumour.

Two major components of past and current breast cancer treatment protocols are chemotherapeutics such as anthracyclines like epirubicin or doxorubicin and ionizing radiation. Their efficiency is based on their strong potential to induce cellular DNA damage. Among other mechanisms, both treatments produce reactive oxygen species (ROS) by iron-mediated oxidation of the doxorubicin quinone structure to a semiquinone radical [5, 6] or by radiation-induced ionization of water [7]. In addition, doxorubicin directly forms radicals via an doxorubicin-iron complex which catalyses the conversion of hydrogen peroxide to hydroxylradicals by repeated redox cycles between Fe (II) and Fe (III) forms [5, 6]. The resulting superoxide radicals, hydrogen peroxides, and hydroxyl radicals quickly react with cellular macromolecules, especially with DNA [8, 9]. The oxidized DNA bases if not removed in time will result in cell cycle arrest and cell death. Thus, the base excision repair (BER) system with its DNA glycosylases specific for various types of oxidative DNA damage is one of the crucial determinants of tumour chemotherapy [10, 11].

Deficiencies in double strand break repair are well described for hereditary and sporadic breast cancer cases [12, 13]. There are also recent reports of genetic variation in BER genes being associated with breast cancer risk [14–18]. Therefore, we hypothesized those single nucleotide polymorphisms (SNPs) in BER genes might contribute to altered DNA repair efficiency, which will affect therapeutic success and cancer specific survival in breast cancer patients. In a prospective breast cancer patient cohort from Germany [19], we assessed whether cancer specific survival is modulated by genetic variation in BER genes according to the therapy applied, especially anthracycline-based chemotherapy and radiotherapy. Although radiotherapy primarily acts on local recurrence, it may nevertheless in consequence have an impact on cancer specific survival [20]. Significant associations were tested for replication in studies of the Breast Cancer Association Consortium (BCAC).

## Methods

### MARIE study population

Breast cancer patients diagnosed at ages 50–74 years between 2001 and 2005 were recruited in the German two-centre (Hamburg and Rhine-Neckar-Karlsruhe region) population-based MARIE study [19] and prospectively followed-up until end of 2009 [21]. The study was approved by the ethics committees of the University of Heidelberg (230/2001 and S-009/2009), the Hamburg Medical Council (1791 and PV3176), and the Medical Board of the State of Rheinland-Pfalz (837.135.09 (6640)) and all participants gave written informed consent.

**Fig. 1:**
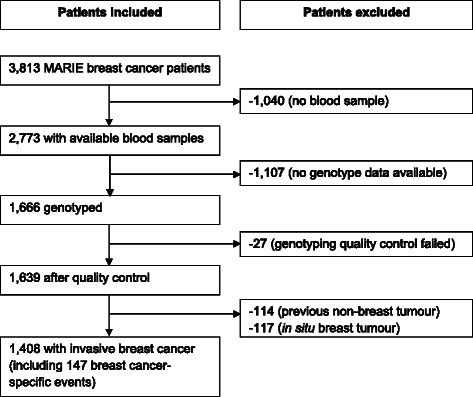
Flowchart on patient selection for survival analysis in the MARIE study

Vital status was assessed via population registries (100 % completeness) and cause of death abstracted from death certificates obtained from the health offices. Of the 3,813 postmenopausal breast cancer patients, genotype information on SNPs in DNA repair genes was available for 1,639 patients. We further excluded patients with previous non-breast tumour (*n* = 114) and with in situ breast tumour (*n* = 117), resulting in 1,408 patients available for this analysis (Fig. 1).

### SNPs selection and genotyping

The initial SNP panel comprised 135 SNPs in 21 base excision repair genes (*APEX1, APEX2, CDKN1A, LIG3, MBD4, MPG, MUTYH, NEIL1, NEIL2, NTHL1, OGG1, PARP1, PARP2, PNKP, POLB, POLG, SMUG1, TDG, TP53, UNG, XRCC1*) [13]. SNPs were mainly common tagging SNPs to capture genetic variation across the genes, plus additional coding SNPs. The SNP selection using HapMap reference data (The International HapMap Consortium 200318; http://www.hapmap.org, HapMap Data Release 22/phase II, NCBI B36 assembly, dbSNP b126) was performed as described previously [15, 22]. Genotyping was conducted using the Illumina GoldenGate Assay. Quality control criteria included barcode labelled plates, 2 % duplicate samples (100 % concordance) and call rates (>96 %). SNPs with poor genotyping clustering were omitted from the analysis [23]. After quality control, genotype data of 127 SNPs in 21 BER genes was available for analysis.

### Statistical analysis of MARIE

Statistical analyses were conducted using SAS 9.2 for MARIE and 9.3 for BCAC data. We used time-to-event analysis (Cox proportional hazards models) to assess the association between genotype and breast cancer specific death, accounting for differences in time between diagnosis and baseline interview date (left truncation: delayed entry models). A log-additive mode of inheritance was assumed for the SNPs.

All models were stratified by age at cancer diagnosis (see Table 1) and study centre (Hamburg and Rhine-Neckar-Karlsruhe region), and adjusted for the following covariates (categorically), obtained by backward selection (*p* <0.05): tumour size, nodal status, baseline metastases status, tumour grade, estrogen/progesterone receptor status, mode of detection, smoking status, menopausal hormone therapy as well as radiotherapy and (anthracycline-based) chemotherapy (including both adjuvant and neoadjuvant treatment).

**Table 1:** Description of the MARIE study population

Characteristics	Overall (*N* = 1,408)	Breast cancer deaths (*N* = 147)
Age at diagnosis		
50–54 years	102 (7.2 %)	11 (7.5 %)
55–59 years	304 (21.6 %)	25 (17.0 %)
60–64 years	446 (31.7 %)	51 (34.7 %)
65–69 years	380 (27.0 %)	38 (25.9 %)
≥70 years	176 (12.5 %)	22 (15.0 %)
Tumour size (cm)		
≤2	774 (55.0 %)	36 (24.5 %)
>2 – ≤5	477 (33.9 %)	65 (44.2 %)
>5	49 (3.5 %)	11 (7.5 %)
Growth into chest wall	43 (3.1 %)	18 (12.2 %)
Neoadjuvant chemotherapy	62 (4.4 %)	16 (10.9 %)
Missings	3 (0.2 %)	1 (0.7 %)
Nodal status (number of affected lymph nodes)^a^		
0	901 (64.0 %)	43 (29.3 %)
1–3	310 (22.0 %)	39 (26.5 %)
4–9	70 (5.0 %)	16 (10.9 %)
≥10	61 (4.3 %)	31 (21.1 %)
Missings	4 (0.3 %)	2 (1.4 %)
Metastasis status		
M0	1356 (96.3 %)	112 (76.2 %)
M1	51 (3.6 %)	34 (23.1 %)
Missings	1 (0.1 %)	1 (0.7 %)
Histological grading^a^		
Grade 1 + 2	963 (68.4 %)	57 (38.8 %)
Grade 3 + 4	376 (26.7 %)	73 (49.7 %)
Missings	7 (0.5 %)	1 (0.7 %)
Hormone receptor status^a^		
ER^+^PR^+^	850 (60.4 %)	60 (40.8 %)
ER^+^PR^−^ or ER^−^PR^+^	271 (19.2 %)	29 (19.7 %)
ER^−^PR^−^	224 (15.9 %)	42 (28.6 %)
Missings	1 (0.1 %)	--
Mode of detection		
Self-detected	794 (56.4 %)	119 (81.0 %)
Routine examination	609 (43.3 %)	28 (19.0 %)
Missings	5 (0.4 %)	--
Radiotherapy		
No	288 (20.5 %)	51 (34.7 %)
Yes	1107 (78.6 %)	94 (63.9 %)
Missings	13 (0.9 %)	2 (1.4 %)
Chemotherapy		
No	718 (51.0 %)	42 (28.6 %)
Yes	675 (47.9 %)	103 (70.1 %)
Anthracycline-based	485 (71.9 %)	74 (71.8 %)
Missings	15 (1.1 %)	3 (2.0 %)
Adult body mass index (BMI)		
≥25 kg/m^2^	360 (25.6 %)	54 (36.7 %)
Missings	--	--
Smoking status		
Never smokers	800 (56.8 %)	83 (56.5 %)
Former smokers	351 (24.9 %)	34 (23.1 %)
Current smokers	257 (18.3 %)	30 (20.4 %)
Menopausal hormone therapy		
Yes, at diagnosis	594 (42.2 %)	34 (23.1 %)
Missings	11 (0.8 %)	3 (2.0 %)

^a^Nodal status, histological grading and hormone receptor status were not determined in the 62 patients who received neoadjuvant chemotherapy (only shown as separate category for tumour size)

We investigated possible differential associations according to chemotherapy overall and anthracycline-based chemotherapy, as well as radiotherapy, using multiplicative interaction terms of SNP * [treatment] (i.e. radiotherapy, chemotherapy, anthracycline-based chemotherapy coded as yes/no). Models with and without interaction term were compared using a likelihood ratio test (LRT). For SNPs with interaction terms showing *p*-value <0.1 in model comparison, stratified analyses according to therapy were conducted to quantify the SNP association with survival according to therapy.

### Replication in the Breast Cancer Association Consortium (BCAC)

SNPs with interaction terms showing *p* <0.1 in the MARIE study were included for replication using studies of BCAC [24]. Data harmonization was applied to all studies in a multi-step process according to a common data dictionary.

Studies were eligible if they had available data on primary invasive breast cancer, genotypes, age, vital status, follow-up, tumour characteristics, and treatment. We restricted the BCAC study population to women aged 50 or older at diagnosis to make it comparable with the postmenopausal MARIE study population. Follow-up time was restricted to 15 years. We further excluded studies with less than ten events, resulting in nine studies (6,392 patients with 526 events) available for this analysis (Additional file 1: Figure S1, Additional file 2: Table S1). All studies were approved by the relevant ethics committees and all participants gave written informed consent.

Genotype data for eight SNPs were available from genotyping conducted using the Illumina iSelect array as part of a large-scale project, the Collaborative Oncological Gene-environment Study (COGS) with thorough centralized quality control measures [24]. Imputed genotypes were available for the other six SNPs using the 1000 genomes project March 2012 release as the reference dataset [25]. The two-stage imputation procedure included the use of SHAPEIT to derive phased genotypes and IMPUTEv2 to perform the imputation on the phased data [26]. Since harmonized individual data were available from the BCAC studies, associations were assessed by pooled analysis using Cox proportional hazard models (allowing for study entry by left truncation) stratified by study and adjusted for tumour stage, tumour grade, ER status, age, principal components to account for population substructure, and radio- and/or chemotherapy.

### Meta-analysis

Meta-analyses were conducted to combine the estimates from the MARIE study and the replication BCAC studies, applying fixed effects models, and to determine study heterogeneity. Study heterogeneity was assessed using I^2^/tau^2^ statistics [27, 28] and forest plots were generated using R (version 2.15.2).

## Results

A description of the patient characteristics of the MARIE study population is provided in Table 1. After a median follow-up time of 72 months (min-max: 3–108 months), 147 patients died from breast cancer and additionally 50 due to other causes. Compared to the total study population, patients who died from breast cancer were more likely to have advanced tumours (larger tumour size, higher nodal status, more often M1 status, poorer grading) and hormone-receptor negative tumours, and more often received chemotherapy and less often radiotherapy.

### Effect modification by chemotherapy

**Fig. 2:**
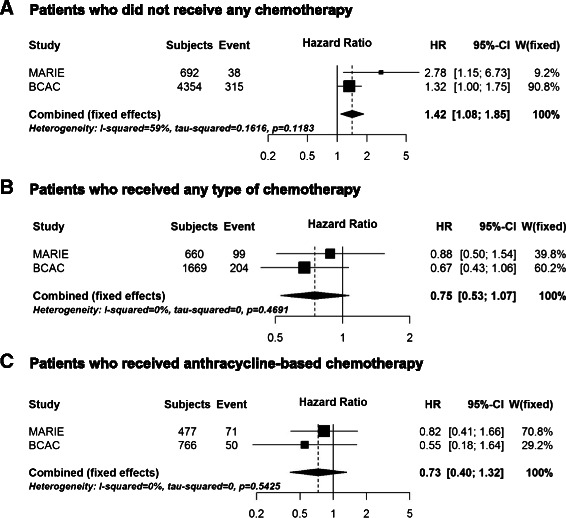
Meta-analysis of *PARP2* rs878156 and breast cancer prognosis according to chemotherapy. Forest plot of of the combined hazard ratios and 95 % confidence intervals for *PARP2* rs878156 in the discovery MARIE study and the replication in Breast Cancer Association Consortium (BCAC) using fixed effect model, according to treatment, i.e. no chemotherapy (**a**), any type of chemotherapy (**b**), and anthracycline-based chemotherapy (**c**). The associations for the BCAC studies were based on pooled analysis stratified by study and adjusted for covariables (see Methods)

**Fig. 3:**
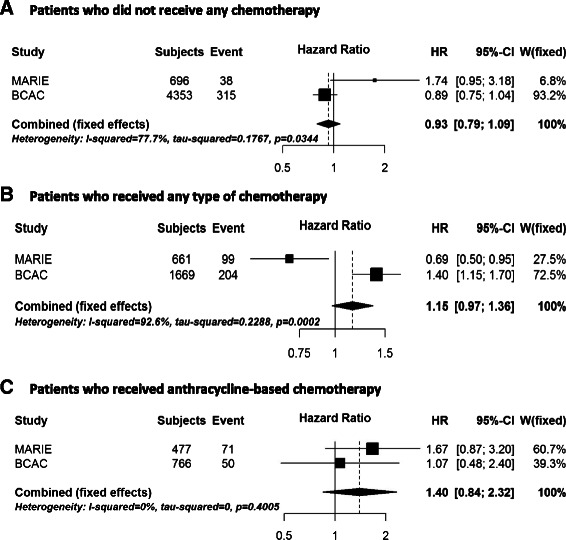
Meta-analysis of *XRCC1* rs3213356 and breast cancer prognosis according to chemotherapy. Forest plot of meta-analysis of hazard ratios and 95 % confidence intervals for *XRCC1* rs3213356 in the discovery MARIE study and the replication in Breast Cancer Association Consortium (BCAC) using fixed effect model, according to treatment, i.e. no chemotherapy (**a**), any type of chemotherapy (**b**) and anthracycline-based chemotherapy . The associations for the BCAC studies were based on pooled analysis stratified by study and adjusted for covariables (see Methods)

In the MARIE study, we identified 14 SNPs in five genes (*OGG1, PARP2, POLB, SMUG1, XRCC1*) with differential effects by any type of chemotherapy (*p* <0.1, Table 2). One SNP in *PARP2* (rs878156) showed a differential association (*p* = 0.093) and was associated with improved survival in MARIE patients who received chemotherapy (HR_chemo_ 0.88, 95 % CI 0.50–1.54) but higher mortality in patients not treated with chemotherapy (HR_no_chemo_ 2.78, 95 % CI 1.15–6.73). The differential association of this SNP with breast cancer specific mortality was successfully replicated in the BCAC studies (*p* = 0.009, HR_chemo_ 0.67, 95 % CI 0.43–1.06 vs. HR_no_chemo_ 1.32, 95 % CI 1.00–1.75). Using a meta-analysis approach, the combined allelic hazard ratios of rs878156 for MARIE and BCAC studies were 0.75 (95 % CI 0.53–1.07) in patients who received chemotherapy and 1.42 (95 % CI 1.08–1.85) in patients not treated with chemotherapy and clearly different (*p* = 0.002) (Fig. 2a, b). There was no evidence of study heterogeneity in a meta-analysis across BCAC studies (Additional file 3: Figure S2). Two SNPs in *XRCC1* showed differential effects in both MARIE and BCAC, however, the SNP associations showed an opposite direction in the BCAC studies to that found in MARIE (Table 2). These two SNPs are in high linkage disequilibrium (*r*^2^ = 0.876). For *XRCC1* rs3213356, we observed significant heterogeneity between the associations observed in the MARIE study and that of the BCAC studies (Fig. 3a, b), confirming the lack of replication, but no study heterogeneity within BCAC studies (Additional file 4: Figure S3).

**Table 2:** Associations between SNP and breast cancer-specific mortality by chemotherapy for interactions showing *p* < 0.1 (LRT)* in the MARIE study and results of replication in BCAC studies

					With chemotherapy			No chemotherapy			
SNP	Alleles	MAF	Gene	Study^a^	HR	95 % CI		HR	95 % CI		*p* for interaction*
rs1052133	C > G	0.22	*OGG1*	MARIE	1.18	0.83	1.66	0.63	0.29	1.37	0.0601
		0.22		BCAC	1.03	0.80	1.32	0.93	0.76	1.14	0.5446
rs2269112	G > A	0.16	*OGG1*	MARIE	1.41	0.97	2.06	0.89	0.40	1.99	0.0498
		0.15		BCAC	1.00	0.76	1.31	1.03	0.82	1.30	0.8695
rs878156	A > G	0.07	*PARP2*	MARIE	0.88	0.50	1.54	2.78	1.15	6.73	0.0930
		0.07		BCAC	0.67	0.43	1.06	1.32	1.00	1.75	0.0093
rs3136717	A > G	0.10	*POLB*	MARIE	1.18	0.74	1.90	0.19	0.05	0.78	0.0388
		0.12		BCAC	0.78	0.55	1.11	0.94	0.74	1.20	0.3787
rs3136781	A > C	0.10	*POLB*	MARIE	1.06	0.64	1.73	0.19	0.05	0.78	0.0599
		0.11		BCAC	0.77	0.54	1.09	0.94	0.74	1.20	0.3583
rs3136790	A > C	0.10	*POLB*	MARIE	1.14	0.70	1.84	0.19	0.05	0.78	0.0474
		0.12		BCAC	0.77	0.54	1.09	0.94	0.74	1.20	0.3452
rs2233921	C > A	0.45	*SMUG1*	MARIE	1.39	1.04	1.87	0.71	0.38	1.32	0.0072
		0.49		BCAC	0.99	0.81	1.21	0.90	0.77	1.06	0.5524
rs2279399	G > A	0.48	*SMUG1*	MARIE	0.79	0.59	1.07	0.99	0.54	1.79	0.0941
		0.44		BCAC	1.01	0.82	1.23	1.08	0.92	1.27	0.7312
rs3087404	G > A	0.48	*SMUG1*	MARIE	0.79	0.59	1.07	1.01	0.55	1.84	0.0874
		0.45		BCAC	1.00	0.82	1.23	1.08	0.92	1.27	0.7083
rs4759344	G > A	0.48	*SMUG1*	MARIE	0.79	0.59	1.07	0.98	0.54	1.79	0.0952
		0.45		BCAC	1.01	0.82	1.23	1.08	0.92	1.27	0.7390
rs6580978	G > A	0.48	*SMUG1*	MARIE	0.79	0.59	1.07	0.99	0.54	1.79	0.0941
		0.45		BCAC	1.01	0.82	1.23	1.08	0.92	1.27	0.7345
rs1799782	G > A	0.06	*XRCC1*	MARIE	1.03	0.59	1.82	0.14	0.02	1.14	0.0965
		0.06		BCAC	1.10	0.73	1.65	1.35	0.97	1.88	0.3074
rs3213255	A > G	0.43	*XRCC1*	MARIE	0.78	0.57	1.08	1.48	0.82	2.69	0.0708
		0.42		BCAC	1.37	1.13	1.67	0.90	0.76	1.06	0.0010
rs3213356	A > G	0.44	*XRCC1*	MARIE	0.69	0.50	0.95	1.74	0.95	3.18	0.0106
		0.44		BCAC	1.40	1.15	1.70	0.89	0.75	1.04	0.0005

*MAF* minor allele frequency, ^a^MARIE: With chemotherapy: 661 (99 events); no chemotherapy: 696 (38 events); BCAC: With chemotherapy: 1,669 (204 events); no chemotherapy: 4,354 (315 events). *P-value for likelihood ratio test (LRT) comparing models with and without the interaction term between SNP and treatment

As the BER system is particularly relevant for oxidative DNA damage due to anthracycline-based chemotherapy, we additionally investigated effect modification by this specific type of chemotherapy, which accounts for about 72 % of chemotherapy regimens. Thirteen SNPs were associated with *p* < 0.1 for breast cancer specific mortality according to anthracycline-based chemotherapy in the MARIE study, nine of them located in the five genes *OGG1, PARP2, POLB, SMUG1, XRCC1* already indicated above and five SNPs in additional three genes (*CDKN1A, LIG3, MBD4*) (Table 3). Solely the *PARP2* SNP rs878156 was consistently associated with improved prognosis after anthracycline-based chemotherapy in both MARIE and BCAC (HR_anthra_ 0.82 and 0.55; *p*_int_ = 0.055 and 0.036, respectively), compared to the poor prognosis for patients without any chemotherapy. The combined allelic HR was 0.73, 95 % CI 0.40–1.32, for the SNP associated survival after anthracycline-based chemotherapy (Fig. 2c), which was not different from that for any chemotherapy but different compared to that for no chemotherapy (Fig. 2a).

**Table 3:** Associations between SNP and breast cancer-specific mortality by anthracycline-based chemotherapy for interactions showing *p* < 0.1 (LRT)* in the MARIE study and results of replication in BCAC studies

					With anthracycline-based chemotherapy			No chemotherapy			
SNP	Alleles	MAF	Gene	Study^a^	HR	95 % CI		HR	95 % CI		*p* for interaction*
rs733590	A > G	0.37	*CDKN1A*	MARIE	0.78	0.52	1.15	1.35	0.75	2.42	0.0781
		0.35		BCAC	1.30	0.87	1.96	1.11	0.94	1.31	0.4857
rs3135989	A > C	0.06	*LIG3*	MARIE	1.67	0.87	3.20	0.84	0.26	2.72	0.0985
		0.07		BCAC	1.07	0.48	2.40	0.95	0.70	1.30	0.8537
rs140697	G > A	0.10	*MBD4*	MARIE	0.39	0.15	0.99	0.89	0.39	2.07	0.0868
		0.09		BCAC	0.93	0.42	2.07	0.84	0.62	1.15	0.9496
rs2005618	A > G	0.10	*MBD4*	MARIE	0.39	0.15	0.99	0.89	0.39	2.07	0.0868
		0.09		BCAC	0.93	0.42	2.07	0.84	0.62	1.15	0.9563
rs1052133	C > G	0.22	*OGG1*	MARIE	1.25	0.85	1.83	0.63	0.29	1.37	0.0687
		0.22		BCAC	0.94	0.55	1.61	0.93	0.76	1.14	0.9496
rs2269112	G > A	0.16	*OGG1*	MARIE	1.43	0.93	2.20	0.71	0.38	1.32	0.0976
		0.15		BCAC	1.14	0.58	2.22	1.03	0.82	1.30	0.8395
rs878156	A > G	0.07	*PARP2*	MARIE	0.82	0.41	1.66	2.78	1.15	6.73	0.0549
		0.07		BCAC	0.55	0.18	1.64	1.32	1.00	1.75	0.0361
rs3136717	A > G	0.10	*POLB*	MARIE	1.42	0.80	2.53	0.19	0.05	0.78	0.0218
		0.12		BCAC	0.45	0.20	1.02	0.94	0.74	1.20	0.0883
rs3136781	A > C	0.10	*POLB*	MARIE	1.45	0.81	2.58	0.19	0.05	0.78	0.0173
		0.11		BCAC	0.45	0.20	1.02	0.94	0.74	1.20	0.0909
rs3136790	A > C	0.10	*POLB*	MARIE	1.45	0.81	2.58	0.19	0.05	0.78	0.0191
		0.12		BCAC	0.45	0.20	1.02	0.94	0.74	1.20	0.0883
rs2233921	C > A	0.45	*SMUG1*	MARIE	1.31	0.92	1.88	0.71	0.38	1.32	0.0156
		0.49		BCAC	0.71	0.47	1.08	0.90	0.77	1.06	0.5463
rs3213255	A > G	0.43	*XRCC1*	MARIE	0.77	0.53	1.12	1.48	0.82	2.69	0.0712
		0.42		BCAC	1.33	0.86	2.04	0.90	0.76	1.06	0.1734
rs3213356	A > G	0.44	*XRCC1*	MARIE	0.73	0.50	1.07	1.74	0.95	3.18	0.0267
		0.44		BCAC	1.20	0.78	1.84	0.89	0.75	1.04	0.2947

*MAF* minor allele frequency, ^a^MARIE: With anthracycline-based chemotherapy: 477 (72 events); no chemotherapy: 696 (38 events); BCAC: With anthracycline-based chemotherapy: 766 (50 events); no chemotherapy: 4,354 (315 events). *P-value for likelihood ratio test (LRT) comparing models with and without the interaction term between SNP and anthracycline treatment

### Effect modification by radiotherapy

Associations were different by radiotherapy (*p* <0.1) for 14 SNPs in five genes (*APEX1, NEIL2, PARP2, TDG, UNG*) in the MARIE study (Additional file 5: Table S2). None of the differential associations were replicated in the BCAC studies.

## Discussion

Using the large cohort of MARIE postmenopausal breast cancer patients for discovery and patient cohorts from studies in BCAC for replication, we found evidence for differential association of rs878156 in the poly (ADP-ribose) polymerase *PARP2* gene with breast cancer specific mortality according to adjuvant chemotherapy. Compared to non-carriers, carriers of the variant G allele experienced improved survival when treated with chemotherapy and poorer survival when they were not treated. A similar effect modification by *PARP2* rs878156 was observed for breast cancer specific mortality after anthracycline-based chemotherapy. To our knowledge, this is the first report of a *PARP2* SNP that is potentially predictive for treatment outcome of anthracycline-based chemotherapy. Studies in breast tumours on associations between PARP2 protein or mRNA expression and prognosis are supportive of our data although results are not conclusive [29, 30].

rs878156 is an intragenic SNP in *PARP2* (minor allele frequency of about 10 %) located 10 base pairs distal from an intron-exon boundary without reported functional impact. Recent research showed that intragenic SNPs which are located even up to 1000 base pairs away from the intron-exon boundary can still affect splicing of the RNA transcript thus modifying protein levels or function [31, 32]. Similar effects are also conceivable for rs878156. This assumption is supported by an increased DNase I sensitivity, high sequence conservation of the SNP region and additional spliced ESTs indicated in the UCSC genome browser (https://genome-euro.ucsc.edu, hg19) but has still to be confirmed experimentally. Regarding PARP2 function, it catalyses, together with PARP1, the poly (ADP-ribosyl) ation of various proteins involved in genome surveillance, especially base excision repair proteins, histones and transcription factors, and in this way modulates the activity of these proteins. Both PARP proteins are induced by DNA-strand interruptions but act on different lesions, such as PARP1 on single-strand breaks or PARP2 on gaps and flap structures [33]. PARP proteins share considerable similarity in the catalytic domain but have different DNA binding domains [33, 34]. There are several inhibitors available affecting both enzymes and some of them are already used in tumour therapy with promising results [35].

As PARP2 contributes to only 5–10 % of the total cellular PARP activity [34], it is difficult to estimate specific PARP2 effects. Therefore, if *PARP2* protein is affected by rs878156, only minor changes are to be expected in normal cells. In case of oxidative damage due to therapy with anthracyclines, however, repair of therapy-related damage might be impaired and therapy efficiency increased. In addition, breast cancer cells frequently harbour genetic or epigenetic modifications that cause DNA repair deficiencies, e.g. mutations or promoter methylation of *BRCA1/2*, *TP53*, *ATM*, *RAD51C, PALB2* [12, 36] or changes in mRNA and protein levels of BER genes [10, 37]. The two repair defects taken together, the tumour-related somatic one and the one caused by the variant germline allele could confer a strong genomic instability to tumour cells, which will increase tumour progression and decrease survival if the patient is not treated. In case of chemotherapy, synthetic lethality could emerge, increasing tumour control by the treatment and thereby improving patient survival, a similar synthetic lethal effect as observed for *BRCA1*-deficient breast tumours treated with PARP inhibitors [11, 13, 38].

Another significant differential association by any chemotherapy was found for the two highly linked *XRCC1* intronic SNPs, rs3213355 and rs3213356, in MARIE. The observed differential association was not formally replicated in the BCAC studies since the direction of the HRs in the subgroups by chemotherapy in BCAC was opposite to that in the MARIE study. Therefore, the observation of differential effects for these two *XRCC1* SNPs in BCAC studies is a new finding, which requires validation in independent studies. Further investigation of genetic variants in *XRCC1* is warranted since a prognostic role of *XRCC1* for breast cancer survival has been reported for the *XRCC1* rs25487 SNP, which causes an amino acid change (e.g. [39–41]). The *XRCC1* variants rs25487 and rs3213356 are not in high linkage disequilibrium. Studies have however reported inconsistent results for the rs25487 risk variant, which could be attributable to investigations in different patient groups with different therapy regimens or focus on patient subgroups like those with metastatic breast cancer.

While the high completeness of follow-up data is a major strength of the MARIE study, our study power to detect weak effects might have been limited with a median follow-up time of only 6 years and 147 events. The effect modulation of therapy response by rs878156 was however confirmed using an independent cohort of more than 6000 breast cancer patients including additional 526 events from BCAC, which demonstrates the robustness of the observed association. As original data collection in the consortium was not standardized and comprehensive across all these studies, we accounted for this limitation through thorough data harmonization and restriction to postmenopausal women aged 50 years and older. In addition, differences in patient characteristics and treatment factors were adjusted for in the statistical analysis to reduce any bias due to study and patient heterogeneity. Although the differential association with rs878156 is not significant if accounting for both the number of SNPs and the different therapies tested, the genes selected were hypothesis driven and thus associated with a high prior probability. Nevertheless, our results should be validated further in clinical studies with homogenous treatment protocols.

## Conclusions

We showed for the first time that the intronic rs878156 SNP in the BER gene *PARP2* can modulate cancer specific survival in breast cancer patients depending on chemotherapy. Thus, if confirmed, this SNP together with further genetic variants that influence prognosis may help to improve treatment decisions in the future. Furthermore, as breast cancer is a heterogeneous disease showing different mutation patterns often involving DNA repair genes, characterization of both tumour and inherited genomes will be required for an improved personalized and targeted treatment.

## Additional files

Additional file 1: Figure S1. Flowchart on sample size for the studies in BCAC used for the replication analysis. (DOCX 16 kb)
Additional file 2: Table S1. Description of the BCAC studies included in this analysis.(DOCX 25 kb)
Additional file 3: Figure S2. Meta-analysis across BCAC studies of *PARP2* and breast cancer prognosis. Forest plot of the combined hazard ratios and 95 % confidence intervals for *PARP2* rs878156 in the discovery MARIE study and the replication studies in Breast Cancer Association Consortium (BCAC) using fixed effect models, according to treatment, i.e. no chemotherapy (A), any type of chemotherapy (B), and anthracycline-based chemotherapy (C). The combined effects for the BCAC studies were also based on fixed effect models. (DOCX 996 kb)
Additional file 4: Figure S3. Meta-analysis across BCAC studies of *XRCC1* and breast cancer prognosis. Forest plot of the combined hazard ratios and 95 % confidence intervals for *XRCC1* rs3213356 in the discovery MARIE study and the replication studies in Breast Cancer Association Consortium (BCAC) using fixed effect models, according to treatment, i.e. no chemotherapy (A), any type of chemotherapy (B), and anthracycline-based chemotherapy (C). The combined effects for the BCAC studies were also based on fixed effect models. (DOCX 1077 kb)
Additional file 5: Table S2. Associations between SNP and breast cancer-specific mortality by radiotherapy for interactions showing *p* <0.1 (LRT)^$^ in the MARIE study and results of replication in BCAC studies. (DOCX 20 kb)

## Abbreviations

BCAC: Breast Cancer Association Consortium
BER: base excision repair
PARP2: poly (ADP-ribose) polymerase 2
*p*_int_: *p*-value for interaction
SNP: single nucleotide polymorphism
