# Supplementary material for: A polymorphism in the base excision repair gene PARP2 is associated with differential prognosis by chemotherapy among postmenopausal breast cancer patients
Source: BMC Cancer. 2015 Dec 16;15:978. doi: 10.1186/s12885-015-1957-7 (PMC4682235; doi:10.1186/s12885-015-1957-7)
Supplement: Additional file 1: Figure S1. — Flowchart on sample size for the studies in BCAC used for the replication analysis. (DOCX 16 kb) [file 12885_2015_1957_MOESM1_ESM.docx]

**Additional file 1**, supplementary Figure S1. Flowchart on sample size for the studies in BCAC used for the replication analysis.

| **Step** |  | **No. of BCAC studies** | **No. of patients** |
| --- | --- | --- | --- |
| 1 | Studies with survival and SNP data in Caucasians | 39 | 47,350 |
| 2 | Delete patients with missing survival and/or follow-up information | 30 | 36,751 |
| 3 | Restrict to first primary breast cancer | 30 | 36,477 |
| 4 | Restrict to invasive breast cancer | 30 | 34,375 |
| 5 | Delete patients younger than 50 years | 30 | 25,235 |
| 6 | Delete the MARIE study | 29 | 23,581 |
| 7 | Delete patients with missing information on age and/or tumour characteristics | 24 | 13,238 |
| 8 | Delete patients with missing information on breast cancer-related death | 24 | 12,809 |
| 9 | Delete patients with missing information on radiotherapy / chemotherapy | 14 | 7,613 |
| 10 | Delete studies with less than 10 events for breast cancer-related death | 9 | 6,392 |

9 BCAC studies available for analysis:

| **Study** | **Total number** | **Events** |
| --- | --- | --- |
| HEBCS | 1014 | 47 |
| KARBAC | 290 | 37 |
| KBCP | 252 | 56 |
| LMBC | 1,634 | 34 |
| NBCS | 514 | 67 |
| RBCS | 96 | 20 |
| SASBAC | 523 | 35 |
| SEARCH | 1,985 | 218 |
| SKKDKFZS | 84 | 12 |
| **all** | **6,392** | **526** |
