# Supplementary material for: A polymorphism in the base excision repair gene PARP2 is associated with differential prognosis by chemotherapy among postmenopausal breast cancer patients
Source: BMC Cancer. 2015 Dec 16;15:978. doi: 10.1186/s12885-015-1957-7 (PMC4682235; doi:10.1186/s12885-015-1957-7)
Supplement: Additional file 2: Table S1. — Description of the BCAC studies included in this analysis.(DOCX 25 kb) [file 12885_2015_1957_MOESM2_ESM.docx]

**Additional file 2, supplementary Table S1.** Description of the BCAC studies included in this analysis.

| **Study Acronym** | **Study Name ^Reference^** | **Country** | **Case Recruitment** |
| --- | --- | --- | --- |
| HEBCS | Helsinki Breast Cancer Study^1,2,3^ | Finland | (1) Consecutive cases (883) from the Department of Oncology, Helsinki University Central Hospital 1997-8 and 2000, (2) Consecutive cases (986) from the Department of Surgery, Helsinki University Central Hospital 2001 – 2004, (3) Familial breast cancer patients (536) from the Helsinki University Central Hospital, Departments of Oncology and Clinical Genetics (1995-). |
| KARBAC | Karolinska Breast Cancer Study^4,5^ | Sweden | 1. Familial cases from Department of Clinical Genetics, Karolinska University Hospital, Stockholm. 2. Consecutive cases from Department of Oncology, Huddinge & Söder Hospital, Stockholm 1998-2000. |
| KBCP | Kuopio Breast Cancer Project^6,7^ | Finland | Women seen at Kuopio University Hospital between 1990-1995 because of a breast lump, mammographic abnormality, or other breast symptom and who were found to have breast cancer. |
| LMBC | Leuven Multidisciplinary Breast Centre^8,9^ | Belgium | All patients diagnosed with breast cancer and seen in the Multidisciplinary Breast Centre in Leuven (Gashuisberg) since June 2007 plus retrospective collection of cases diagnosed since 2000. |
| NBCS | Norwegian Breast Cancer Study^10^ | Norway | Incidence cases from three different hospitals: Ullevål Univ. Hospital 1990-94, Norwegian Radium Hospital 1975-1986 and 1995-1998, Haukeland Univ. Hospital 1992-2001. |
| RBCS | Rotterdam Breast Cancer Study^11^ | Netherlands | Familial breast cancer patients selected from the clinical genetics centre at Erasmus Medical Centre between 1994 and 2005. |
| SASBAC | Singapore and Sweden Breast Cancer Study^12^ | Sweden | Women diagnosed in Sweden aged 50-74 in 1993-1995. |
| SEARCH | Study of Epidemiology & Risk Factors in Cancer Heredity^13^ | U.K. | Identified through the Eastern Cancer Registration and Information Centre: (i) prevalent cases; diagnosed 1991-1996; under 55 years of age at diagnosis; recruited 1996-2002 (ii) incident cases; diagnosed since 1996; under 70 years of age at diagnosis; recruited 1996-present. |
| SKKDKFZS | Städtisches Klinikum Karlsruhe Deutsches Krebsforschungszentrum Study^14^ | Germany | Women diagnosed with primary *in situ* or invasive breast cancer at the Städtisches Klinikum Karlsruhe from March 1993 to July 2005. Cases were 21-93 years of age. |

1) Syrjakoski K et al. (2000) Population-based study of BRCA1 and BRCA2 mutations in 1035 unselected Finnish breast cancer patients. J Natl Cancer Inst 92:1529-31.

2) Kilpivaara O et al. (2005) Correlation of CHEK2 protein expression and c.1100delC mutation status with tumor characteristics among unselected breast cancer patients. Int J Cancer 113:575-80.

3) Fagerholm R et al. (2008) NAD(P)H:quinone oxidoreductase 1 NQO1*2 genotype (P187S) is a strong prognostic and predictive factor in breast cancer. Nat Genet 40:844-853.

4) Lindblom A et al. (1992) Hereditary breast cancer in Sweden: a predominance of maternally inherited cases. Breast Cancer Res Treat 24:159-65.

5) Margolin S et al. (2004) BRCA1 mutations in a population –based study of breast cancer in Stockholm County. Genet Test:127-32.

6) Hartikainen JM et al. (2005) An autosome-wide scan for linkage disequilibrium-based association in sporadic breast cancer cases in eastern Finland: three candidate regions found. Cancer Epidemiol Biomarkers Prev 14:75-80.

7) Hartikainen JM et al. (2006) Refinement of the 22q12-q13 breast cancer-associated region: evidence of TMPRSS6 as a candidate gene in an eastern Finnish population. Clin Cancer Res 12:1454-1462.

8) Neven P et al. (2008) In early-stage breast cancer, the estrogen receptor interacts with correlation between human epidermal growth factor receptor 2 status and age at diagnosis, tumor grade, and lymph node involvement. J Clin Oncol.26(10):1769-71.

9) De Maeyer L et al. (2008) Does estrogen receptor negative/progesterone receptor positive breast carcinoma exist? J Clin Oncol 26(2):336-8.

10) Zheng W et al. (2009) Genome-wide association study identifies a new breast cancer susceptibility locus at 6q25.1. Nature Genetics 41(3):324-8

11) Easton DF et al. (2007) Genome-wide association study identifies novel breast cancer susceptibility loci. Nature 447: 1087-1093 (2007).

12) Wedren S et al. (2004) Oestrogen receptor alpha gene haplotype and postmenopausal breast cancer risk: a case control study. Breast Cancer Res 6:R437-49.

13) Lesueur F. et al. (2005) Allelic association of the human homologue of the mouse modifier Ptprj with breast cancer. Hum Mol Genet 14:2349-56.

14) Stevens KN et al. (2012) 19p13.1 is a triple-negative-specific breast cancer susceptibility locus. Cancer Res 72(7):1795-803.
