# Supplementary material for: A polymorphism in the base excision repair gene PARP2 is associated with differential prognosis by chemotherapy among postmenopausal breast cancer patients
Source: BMC Cancer. 2015 Dec 16;15:978. doi: 10.1186/s12885-015-1957-7 (PMC4682235; doi:10.1186/s12885-015-1957-7)
Supplement: Additional file 4: Figure S3. — Meta-analysis across BCAC studies of XRCC1 and breast cancer prognosis. Forest plot of the combined hazard ratios and 95 % confidence intervals for XRCC1 rs3213356 in the discovery MARIE study and the replication studies in Breast Cancer Association Consortium (BCAC) using fixed effect models, according to treatment, i.e. no chemotherapy (A), any type of chemotherapy (B), and anthracycline-based chemotherapy (C). The combined effects for the BCAC studies were also based on fixed effect models. (DOCX 1077 kb) [file 12885_2015_1957_MOESM4_ESM.docx]

1. **Patients who did not receive any chemotherapy**

**
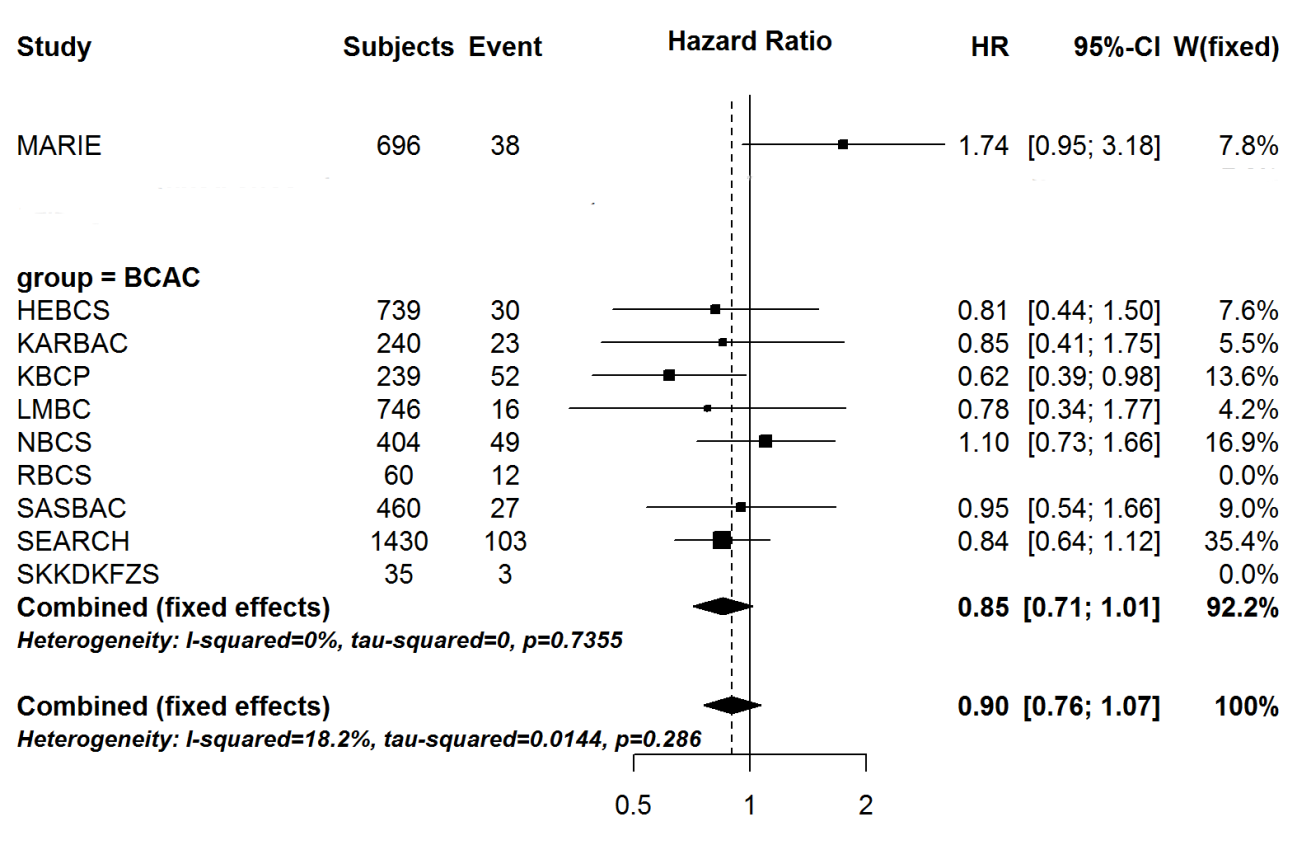
**

1. **Patients who received any type of chemotherapy**

**
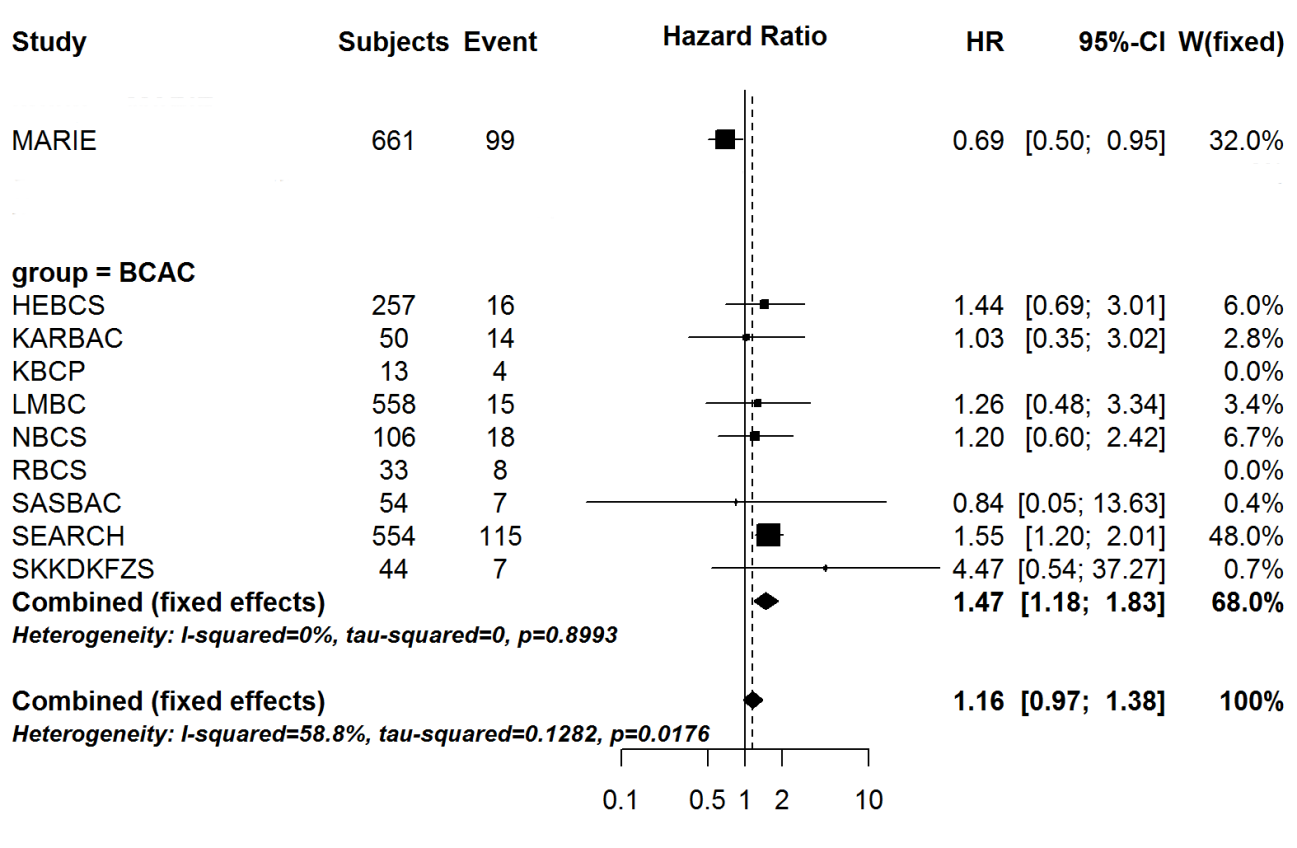
**

1. **Patients who received anthracycline-based chemotherapy**

**
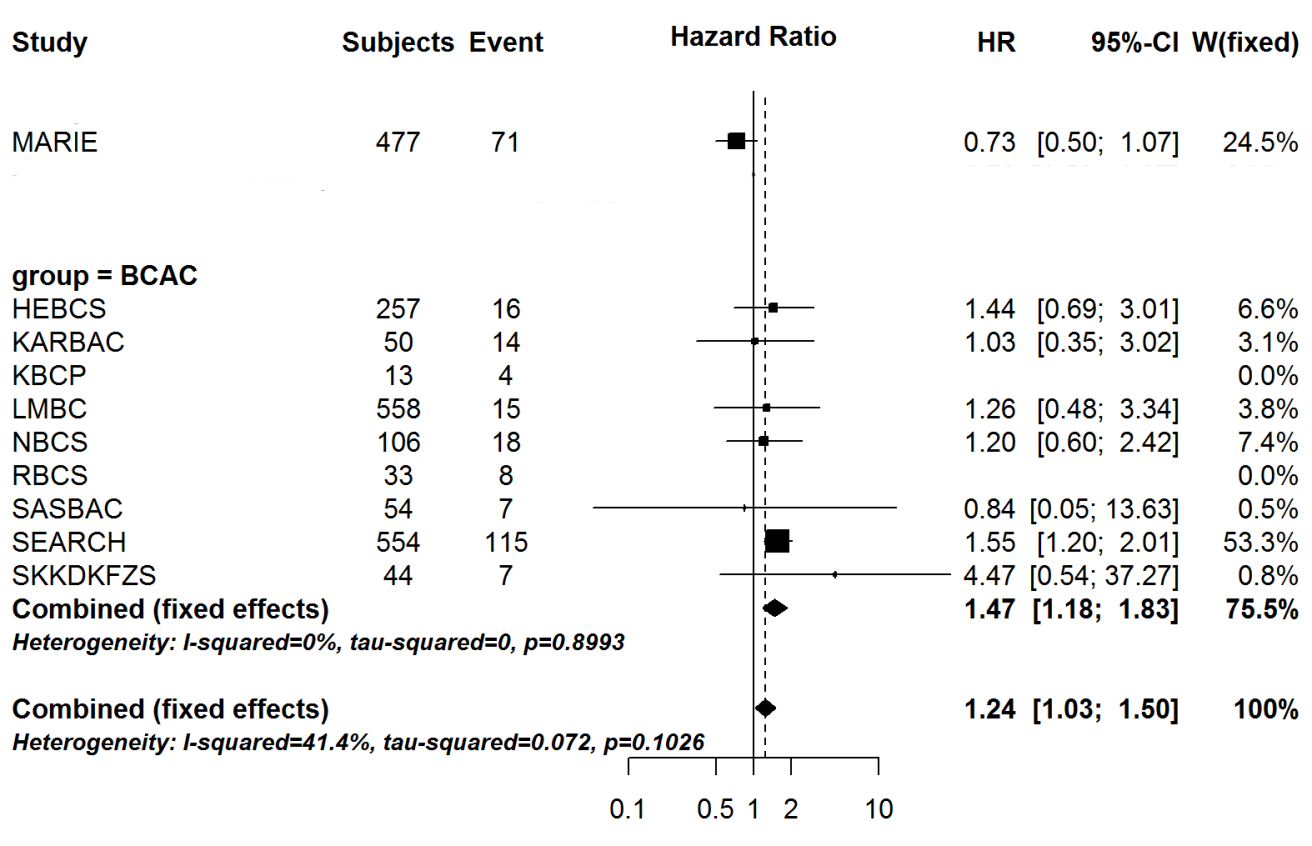
**

**Additional file 4, supplementary Figure 3. Meta-analysis of *XRCC1* rs3213356 and breast cancer prognosis.** Forest plot of the combined hazard ratios and 95% confidence intervals for *XRCC1* rs3213356 in the discovery MARIE study and the replication studies in Breast Cancer Association Consortium (BCAC) using fixed effect models, according to treatment, i.e. no chemotherapy (A), any type of chemotherapy (B), and anthracycline-based chemotherapy (C). The combined effects for the BCAC studies were also based on fixed effect models.
