# Supplementary material for: A polymorphism in the base excision repair gene PARP2 is associated with differential prognosis by chemotherapy among postmenopausal breast cancer patients
Source: BMC Cancer. 2015 Dec 16;15:978. doi: 10.1186/s12885-015-1957-7 (PMC4682235; doi:10.1186/s12885-015-1957-7)
Supplement: Additional file 5: Table S2. — Associations between SNP and breast cancer-specific mortality by radiotherapy for interactions showing p <0.1 (LRT)$ in the MARIE study and results of replication in BCAC studies. (DOCX 20 kb) [file 12885_2015_1957_MOESM5_ESM.docx]

**Additional file 5, Supplementary Table S2.** Associations between SNP and breast cancer-specific mortality by radiotherapy for interactions showing p <0.1 (LRT)^$^ in the MARIE study and results of replication in BCAC studies

|  |  |  |  |  | **With radiotherapy** | | |  | **No radiotherapy** | | |  |  |
| --- | --- | --- | --- | --- | --- | --- | --- | --- | --- | --- | --- | --- | --- |
| **SNP** | **Alleles** | **MAF** | **Gene** | **Study*** | **HR** | **95% CI** | |  | **HR** | **95% CI** | |  | **p for interaction**^$^ |
| rs1320150 | A>G | 0.43 | *APEX1* | MARIE | 0.77 | 0.56 | 1.07 |  | 1.22 | 0.75 | 2.01 |  | 0.0826 |
|  |  | 0.41 |  | BCAC | 0.98 | 0.84 | 1.14 |  | 0.73 | 0.56 | 0.95 |  | 0.0950 |
| rs8191589 | T>A | 0.20 | *NEIL2* | MARIE | 0.82 | 0.55 | 1.23 |  | 1.84 | 0.96 | 3.53 |  | 0.0643 |
|  |  | 0.21 |  | BCAC | 1.07 | 0.90 | 1.27 |  | 0.79 | 0.57 | 1.11 |  | 0.0673 |
| rs2297616 | G>A | 0.27 | *PARP2* | MARIE | 0.76 | 0.54 | 1.08 |  | 1.49 | 0.86 | 2.58 |  | 0.0306 |
|  |  | 0.27 |  | BCAC | 1.04 | 0.88 | 1.23 |  | 0.94 | 0.72 | 1.23 |  | 0.5302 |
| rs3093904 | T>A | 0.27 | *PARP2* | MARIE | 0.76 | 0.54 | 1.08 |  | 1.38 | 0.80 | 2.40 |  | 0.0490 |
|  |  | 0.27 |  | BCAC | 1.04 | 0.88 | 1.23 |  | 0.94 | 0.72 | 1.23 |  | 0.5297 |
| rs7159947 | A>G | 0.36 | *PARP2* | MARIE | 0.85 | 0.62 | 1.16 |  | 1.47 | 0.90 | 2.40 |  | 0.0689 |
|  |  | 0.36 |  | BCAC | 1.04 | 0.89 | 1.22 |  | 1.06 | 0.83 | 1.36 |  | 0.9748 |
| rs878157 | G>A | 0.28 | *PARP2* | MARIE | 0.76 | 0.53 | 1.07 |  | 1.32 | 0.77 | 2.28 |  | 0.0707 |
|  |  | 0.26 |  | BCAC | 1.08 | 0.92 | 1.27 |  | 0.92 | 0.71 | 1.20 |  | 0.3067 |
| rs2629768 | G>A | 0.15 | *TDG* | MARIE | 1.12 | 0.74 | 1.70 |  | 0.40 | 0.18 | 0.89 |  | 0.0758 |
|  |  | 0.16 |  | BCAC | 1.04 | 0.84 | 1.29 |  | 0.80 | 0.55 | 1.14 |  | 0.1531 |
| rs2723876 | A>C | 0.15 | *TDG* | MARIE | 1.12 | 0.74 | 1.70 |  | 0.40 | 0.18 | 0.89 |  | 0.0758 |
|  |  | 0.14 |  | BCAC | 1.04 | 0.84 | 1.30 |  | 0.80 | 0.55 | 1.14 |  | 0.1469 |
| rs322105 | C>G | 0.15 | *TDG* | MARIE | 1.12 | 0.74 | 1.70 |  | 0.40 | 0.18 | 0.89 |  | 0.0763 |
|  |  | 0.16 |  | BCAC | 1.04 | 0.84 | 1.29 |  | 0.80 | 0.55 | 1.14 |  | 0.1533 |
| rs322106 | A>G | 0.15 | *TDG* | MARIE | 1.10 | 0.73 | 1.67 |  | 0.40 | 0.18 | 0.88 |  | 0.0819 |
|  |  | 0.16 |  | BCAC | 1.04 | 0.84 | 1.29 |  | 0.80 | 0.55 | 1.14 |  | 0.1533 |
| rs322107 | G>A | 0.15 | *TDG* | MARIE | 1.12 | 0.74 | 1.70 |  | 0.40 | 0.18 | 0.89 |  | 0.0763 |
|  |  | 0.16 |  | BCAC | 1.04 | 0.84 | 1.29 |  | 0.80 | 0.55 | 1.14 |  | 0.1533 |
| rs4135036 | A>G | 0.08 | *TDG* | MARIE | 1.21 | 0.71 | 2.06 |  | 0.58 | 0.20 | 1.67 |  | 0.0944 |
|  |  | 0.08 |  | BCAC | 0.80 | 0.59 | 1.08 |  | 0.92 | 0.59 | 1.42 |  | 0.6697 |
| rs4135128 | G>C | 0.11 | *TDG* | MARIE | 1.35 | 0.83 | 2.18 |  | 0.73 | 0.29 | 1.82 |  | 0.0619 |
|  |  | 0.10 |  | BCAC | 0.87 | 0.67 | 1.12 |  | 0.96 | 0.65 | 1.42 |  | 0.7379 |
| rs2569987 | A>G | 0.18 | *UNG* | MARIE | 0.68 | 0.44 | 1.06 |  | 1.79 | 0.94 | 3.41 |  | 0.0063 |
|  |  | 0.17 |  | BCAC | 1.02 | 0.84 | 1.23 |  | 1.14 | 0.84 | 1.55 |  | 0.8719 |

MAF, Minor allele frequency; * MARIE: With radiotherapy: 1,085 (91 events); no radiotherapy: 272 (46 events); BCAC: With radiotherapy: 4,520 (369 events); no radiotherapy: 1,503 (150 events) ^$^ P value for likelihood ratio test (LRT) comparing models with and without the interaction term between SNP and radiotherapy
